# Improved Heterosis Prediction by Combining Information on DNA- and Metabolic Markers

**DOI:** 10.1371/journal.pone.0005220

**Published:** 2009-04-16

**Authors:** Tanja Gärtner, Matthias Steinfath, Sandra Andorf, Jan Lisec, Rhonda C. Meyer, Thomas Altmann, Lothar Willmitzer, Joachim Selbig

**Affiliations:** 1 Department of Bioinformatics, Institute of Biochemistry and Biology, University of Potsdam, Potsdam-Golm, Germany; 2 Bioinformatics and Biomathematics Group, Genetics and Biometry Unit, Research Institute for the Biology of Farm Animals (FBN), Dummerstorf, Germany; 3 Max-Planck-Institute of Molecular Plant Physiology, Potsdam-Golm, Germany; 4 Department of Molecular Biology, Leibniz Institute of Plant Genetics and Crop Plant Research (IPK), Gatersleben, Germany; University of Massachusetts Amherst, United States of America

## Abstract

**Background:**

Hybrids represent a cornerstone in the success story of breeding programs. The fundamental principle underlying this success is the phenomenon of hybrid vigour, or heterosis. It describes an advantage of the offspring as compared to the two parental lines with respect to parameters such as growth and resistance against abiotic or biotic stress. Dominance, overdominance or epistasis based models are commonly used explanations.

**Conclusion/Significance:**

The heterosis level is clearly a function of the combination of the parents used for offspring production. This results in a major challenge for plant breeders, as usually several thousand combinations of parents have to be tested for identifying the best combinations. Thus, any approach to reliably predict heterosis levels based on properties of the parental lines would be highly beneficial for plant breeding.

**Methodology/Principal Findings:**

Recently, genetic data have been used to predict heterosis. Here we show that a combination of parental genetic and metabolic markers, identified via feature selection and minimum-description-length based regression methods, significantly improves the prediction of biomass heterosis in resulting offspring. These findings will help furthering our understanding of the molecular basis of heterosis, revealing, for instance, the presence of nonlinear genotype-phenotype relationships. In addition, we describe a possible approach for accelerated selection in plant breeding.

## Introduction

The introduction of the concept of hybrids probably represents the most important single innovation in plant breeding. Central to the concept of hybrid breeding is the phenomenon of heterosis or hybrid vigour [1], which essentially describes the superiority of the hybrid line derived from two parental inbred lines with respect to numerous parameters, with yield being the most important one. It is crucial to note that the superiority of the hybrid is not only realized in comparison to the two parental lines but most importantly also in comparison to lines obtained by classical line breeding [2]. Crops strongly relying on hybrid breeding include maize [2], rye [3], sugar beet [4], rice [5] or oilseed rape [6], [7].

Despite its central importance for the hybrid breeding concept, the molecular basis responsible for the heterosis effects is far from being understood. From a genetics perspective, dominance, overdominance or epistasis based models are commonly used as explanations for heterosis [8].

One central observation in hybrid breeding programs is that the extent of heterosis is strongly dependent on the two parental lines. Thus, with respect to yield, for instance, heterosis can vary strongly depending upon the specific combination. In addition to its puzzling biological complexity, this represents a severe challenge to plant breeding, as usually the best combination of two parental lines can only be determined by trial and error. Therefore many thousands of testcrosses are required in order to find the optimal parental combination for the trait of interest. Numerous attempts have been followed with the goal to reduce the level of uncertainty concerning suitable parental combinations and to achieve some level of predictability. Not surprisingly mostly genetic markers of the two parents were used in these approaches [9]–[12].

Despite the fact that genetic markers have proven to be highly useful in plant breeding for marker-assisted selection of simple traits that are difficult to assay [13]–[15], they have their limitations when it comes to complex phenomenons involving many genes, such as heterosis. Even using dense genetic maps, marker intervals can still cover several hundred genes [16], i.e. their genetic resolution is low and their ability to account for complex interactions between several or many genes and their products is limited.

We set out to test whether heterosis prediction can be improved by using more complex parameters of the parental line than genetic markers. To this end, we decided to investigate parental metabolic markers, i.e. relative levels of particular metabolic compounds, with regard to their predictive power for biomass heterosis in the plant model organism *Arabidopsis thaliana*. The reason for choosing these predictors is that metabolite levels are the result of more genes than those represented by genetic markers, and as a rule they are also influenced by several genes and/or their products.

As shown in the Results section, the predictive power of genetic data for heterosis is significantly improved by combining it with metabolic measurements of one parent, suggesting complex mechanisms underlying heterosis. Finally, when analyzing the minimal set of metabolic and genetic markers needed for heterosis prediction in the two different testcross populations used, three classes can be identified. Firstly, there are markers highly predictive in both kinds of testcross combinations, secondly, we find markers specifically predictive for one kind of testcross combination, and there are also markers negligible in any of our prediction models.

## Results

### Design of the experiment and analysis

As described in the Introduction, heterosis levels are a function of the two parental lines used to produce the offspring. Classical heterosis experiments are performed between two completely different parental lines, which have been inbred to a high degree and are essentially homozygous, resulting in a highly heterozygous F1 offspring. We here deliberately chose a different and genetically simpler experimental set-up as a first step towards investigation of the information carried by metabolic markers in comparison to, or in combination with, molecular genetic markers. To this end, a recombinant inbred line (RIL) population consisting of 359 lines derived from a cross between the two *Arabidopsis thaliana* accessions C24 and Col-0 [17] was used. The mean frequency of C24 alleles and Col-0 alleles per RIL was 48% and 50%, respectively. The remaining fraction comprised heterozygous regions and a few missing values (cf. Methods section for details). The 359 RILs served as one parental population. They were backcrossed with both, the C24 and the Col-0 accessions, resulting in a total of 718 testcross-progeny.

**Figure 1:**
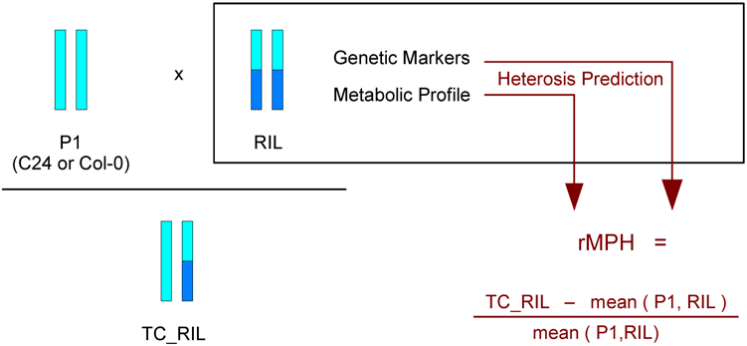
Rationale of the experimental outline. The experimental set-up comprised the two *Arabidopsis thaliana* accessions C24 and Col-0, which served one at a time as the first parent of the testcrosses. One of 359 recombinant inbred lines derived from the two original accessions acted as the second parent. The analysis implicated the metabolic profile and genetic markers of the RILs, which were then used to predict the relative mid-parent heterosis rMPH in biomass. The latter is defined as the relative biomass gain of the testcross as compared to the mean biomass of its parents (cf. Methods section for details) and therefore manifests itself not until the next generation. P1 describes the shoot biomass of the first parent, RIL the shoot biomass of the particular recombinant inbred line and TC_RIL the shoot biomass of the corresponding testcross. The function mean(•, •) refers to the arithmetic mean of the respective values.

The analysis of a full or half diallele involves crosses among numerous lines with different degrees of relatedness, and in each cross only a subset of the genetic factors responsible for heterosis might be shared with any other cross. In contrast to that, in our much simpler experimental design one parent was kept constant (C24 and Col-0 respectively, cf. Figure 1). Consequently, we were able to use prediction models which consider just the metabolic/genetic markers of the particular RIL parent, since those of the second parent are constants. We merely had to distinguish the two different response effects C24-heterosis and Col-heterosis, i.e. the relative biomass gain when a RIL is backcrossed to C24 and Col-0, respectively. In that way, we were able to detect not only markers for heterosis prediction, but also factors which are involved in heterosis in only one particular testcross setting, for instance in Col-0 testcrosses. Such Col-heterosis specific markers hold the potential to reveal loci with dominance for C24 or overdominance effects, whereas C24-heterosis specific markers indicate dominance for Col-0 or overdominance.

To determine the degree of heterosis displayed in the two different testcross set-ups, biomass was measured in the testcross-progeny and compared to the mean of the biomasses of the two corresponding parental lines (cf. Figure 1). Thus the relative mid parent heterosis was observed to vary from approximately −36 per cent to 99 per cent depending on the parental lines used. Analyses of the genetic markers and the metabolic profile of the RIL population have been described previously [17]–[19].

For training and evaluating the prediction models, we applied a partial least squares (PLS) approach [20], using a leave-one-out validation (LOOV) test (cf. Methods section for details).

### Using all available genetic or metabolic markers for heterosis prediction leads to an overfitting problem

In a first approach we compared the whole set of available genetic versus metabolic markers according to their suitability for heterosis prediction. Applying PLS-modelling on all 110 genetic markers, the Pearson correlation between observed and in cross-validation predicted heterosis was 0.39 for the C24 crosses and 0.35 for the Col-0 crosses. Using, on the other hand, all available 181 metabolic markers as predictor set, the corresponding numbers were 0.27 for C24 crosses and 0.24 for Col-0 testcrosses.

These results show that some prediction has been achieved. But the level of predictive power is very low, above all in the case of the 181 metabolic markers. We believe this to be a problem of overfitting, indicated by the fact that the larger predictor set yields the smaller prediction success in cross-validation.

### Feature selection overcomes the problem of overfitting and leads to an almost equal predictive power of genetic and metabolic predictors

In order to rank the available variables according to their contribution to the response, we utilized the variable importance in the projection (VIP), which calculates the contribution of each predictor variable to the response in the respective PLS-model [20], [21]. To reduce the number of variables in the respective models we kept only those with a high contribution. We determined the threshold via optimizing the predictive power in LOOV. Subsequently the reduced model that resulted in the highest predictive power was selected.

**Figure 2:**
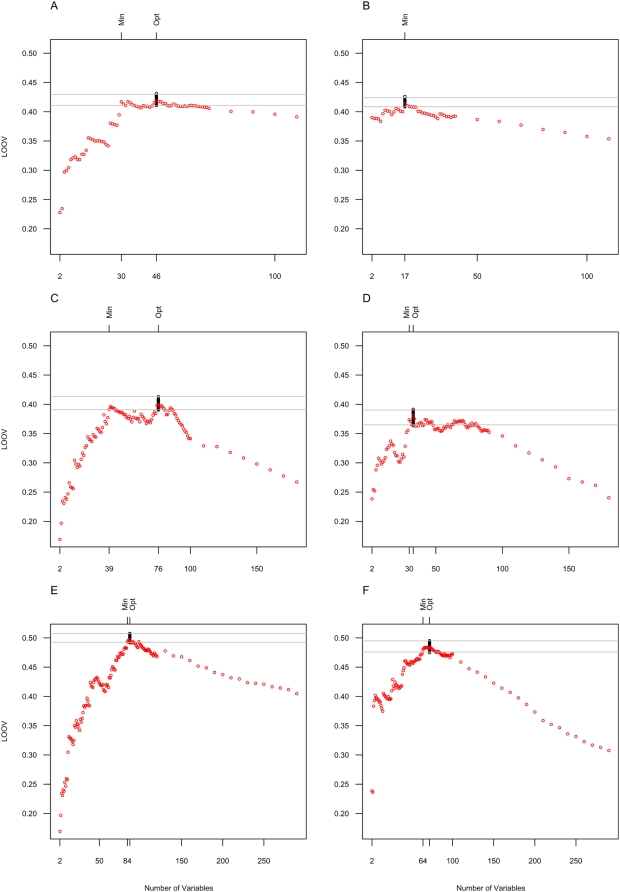
Predictive power of genetic (A, B), metabolic (C, D) and combined (E, F) marker sets for C24- and Col-heterosis. The diagrams shown here demonstrate the trade-off between overfitting and loss of information in the different models. The x-axis represents the number of predictors used to train the respective model. Red dots display the predictive power of the particular model in leave-one-out validation (LOOV). Panel A, C and E correspond to C24-heterosis, the remaining ones to Col-heterosis. The number of predictors, which maximizes the predictive power, is referred to as Opt, and it differs in the various models. In each case, the predictive power decreases by incorporating too many predictors in the corresponding model. This effect is due to overfitting. On the other hand, loss of information occurs, if too few predictors are selected in the model. Min refers to the minimal number of predictors that does not yet imply a significant loss of predictive power. The corresponding predictive power is still within the estimated confidence interval (gray lines) of the maximal predictive power. Black dots demonstrate the estimation of these confidence intervals. They represent the predictive power of the optimal predictor set when using jackknife resampled data (cf. Methods section for details).

Applying this feature selection approach to our four PLS models (i.e. heterosis according to the Col-0 or C24 parent using either metabolic or genetic markers) led to improvements of the predictive power to values of about 0.40 (cf. Table 1), irrespective of the use of exclusively genetic or exclusively metabolic markers. Due to the different degree of the overfitting problem, improvements were more substantial in case of metabolic as compared to genetic markers resulting in a nearly equal predictive power of both marker sets (cf. Figure 2 A–D and Table S1).

**Table 1:** Predictive power (PP) in leave-one-out validation of the respectively optimal selections of predictors for the relative mid-parent heterosis regarding the two different testcross set-ups.

Response variable	C24-heterosis	Col-heterosis
**PP of optimal genetic selection**	0.42	0.41
**PP of optimal metabolic selection**	0.40	0.38
**PP of optimal combined genetic-metabolic selection**	0.50	0.48

### Combining metabolic and genetic markers leads to substantial improvements of heterosis prediction

We wanted to answer the question of whether a combination of genetic and metabolic markers improves the predictability of heterosis as compared to the use of genetic markers alone. Since our variable selection algorithm allowed to overcome the problem of overfitting when dealing with a high number of predictors, we were now in a position to apply this algorithm on the combined set of metabolic and genetic markers (i.e. a total of 291 predictors) and test their suitability for heterosis prediction using PLS regression (cf. Figure 2).

As shown in Table 1, the combination of metabolic and genetic markers leads to a strong improvement of the predictive power in LOOV as compared to the genetic or metabolic marker based models. The improvement was highly significant in each case, since the estimated confidence intervals of the respective correlation values did not overlap (cf. Table S1).

### Marker identification via minimum-description-length (MDL) based strategies

One advantage of the experimental design used here is the identification of variables that are important for heterosis prediction in both testcross populations as compared to variables that are strongly relevant in the prediction models for only one kind of combination, i.e. either with C24 or with Col-0. However, in order to perform this analysis, the number of variables in the respective models had to be reduced further to a minimal set of variables whose predictive power does not differ significantly from the optimal predictive power. To achieve this goal we developed a MDL based strategy (cf. Methods section). Application of this approach to the single metabolic models, to the genetic models and to the combined models led to a substantial reduction in the number of variables needed, as compared to the number of measured variables, in each case (cf. Table S2 and Figure 2). Thus, 68 out of the 110 available genetic and 98 out of the 181 available metabolic markers were omitted in each of the corresponding four minimal models investigated without any significant loss of predictive power, as compared to the optimally achievable predictive power in the particular predictor set. This means that more than half of the markers measured turned out to be of no significant relevance for heterosis prediction in our analysis, neither in the case of C24 nor in the case of Col-0 testcrosses (cf. Table S3).

### Genetic markers important as heterosis predictors overlap with heterosis quantitative trait loci (QTL)

**Figure 3:**
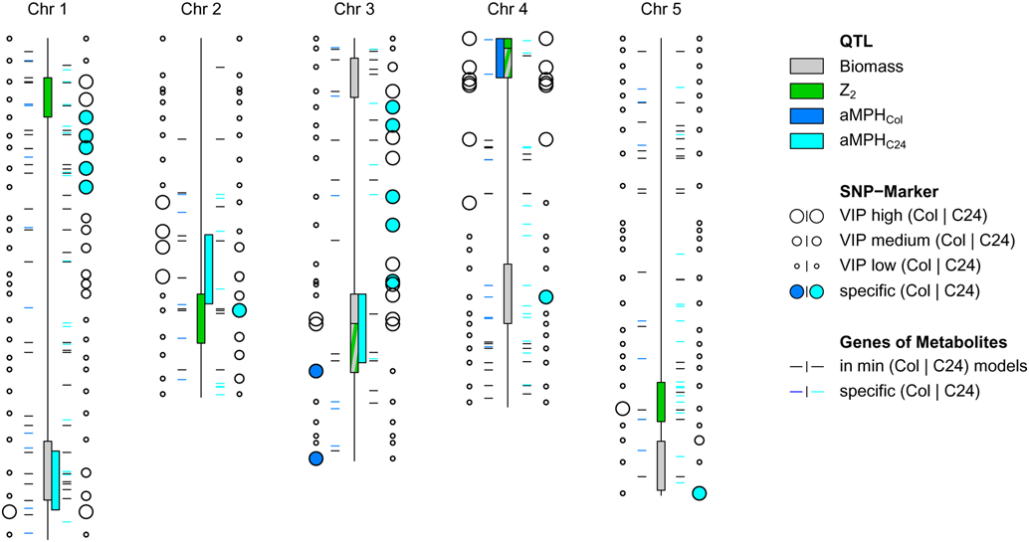
Discrete VIP of genetic markers for Col-heterosis and C24-heterosis prediction, including overlap with QTL. Each of the 110 genetic markers and its position on a Chromosome (Chr) is represented by two circles. The circle size indicates high (large circles), medium and low (small circles) VIP in the genetic model for Col-heterosis (circle left of chromosome) and C24-heterosis prediction (circle right of chromosome). We refer to the VIP as high and medium, if the corresponding marker is contained in the minimal genetic model and in the optimal genetic model, respectively. Markers specific for Col-heterosis prediction and those specific for C24-heterosis prediction are coloured in dark blue and in light blue, respectively. To allow positional comparison, support intervals of biomass QTL and three kinds of heterosis related QTL detected by Meyer et al. (2008, submitted) are plotted as coloured boxes along the chromosomes (grey: biomass, green: Z2 [40], dark blue: absolute mid-parent heterosis concerning Col-0, referred to as aMPH_Col, light blue: absolute mid-parent heterosis concerning C24, referred to as aMPH_C24). Horizontal lines represent the position of genes directly involved in reactions including metabolites, which are contained in the respective minimal metabolite model and the combined genetic-metabolic model (Col: left of chromosomes, C24: right of chromosomes). For specific metabolites genes were coloured accordingly.

When comparing the relative importance of the 110 genetic markers as judged by their contribution to heterosis prediction with the genetic analysis of heterotic biomass QTL, a substantial overlap is observed (cf. Figure 3). In particular this is true for the following markers that bear a special meaning.

Eight genetic markers turned out to be highly predictive for heterosis in both testcross populations. This was indicated by their presence in the minimal genetic model and in the minimal combined genetic-metabolic model for both, C24- and Col-heterosis prediction. Five of those highly predictive markers are located at the top of chromosome 4, a region containing important QTL for heterosis and biomass *per se* [22], cf. Meyer *et al.* (2008 submitted). The same is true for the two highly predictive markers at chromosome 3. The remaining one important marker at bottom of chromosome 1 also coincides with one heterotic QTL (cf. Figure 3).

Fifteen genetic markers are specific for predicting heterosis in either the Col-0 or the C24 crosses. They are contained in the minimal genetic model and in the minimal combined genetic-metabolic model for one testcross population, but in none of the corresponding models for the other testcross population. Two of these markers are specific for Col-heterosis prediction. They are both located at the bottom of chromosome 3, while the thirteen markers specific for C24-heterosis prediction are distributed to all five chromosomes. Also in the case of the specific genetic markers there is some overlap with QTL (cf. Figure 3).

### Identified metabolic markers deviate from normal distribution

Applying the MDL based feature selection method to the metabolic markers, we identified the most important ones with respect to heterosis prediction (cf. Tables S4 and S5). Sixteen of them are contributing to the minimal models only in the C24 testcrosses, while ten metabolic markers in our models are of specific importance with respect to Col-heterosis prediction (cf. Table 2).

**Table 2:** Metabolic markers highly predictive (pred) in both testcross (TC) populations and those specifically for heterosis (het) prediction in one certain testcross set-up, each in alphabetical order.

Highly pred in both TC set-ups	Specific for C24-het prediction	Specific for Col-het prediction
Alanine	Fumaric acid	Gluconic acid
Cellobiose	Galactose	Glycerol-3-phosphate
Glycine	Glucose-6-phosphate	Fructose-6-phosphate
Oxalic acid	Maleic acid	2,4-Hydroxy butanoic acid
Propanoic acid	Maltose	α-Tocopherol
Urea	Putrescine	5 Unknowns
7 Unknowns	Raffinose	
	Salicylic acid	
	Tyrosine	
	7 Unknowns	

On the other hand, fourteen metabolic markers were identified as important for heterosis prediction irrespective of the parent (cf. Table 2). Five of the latter display metabolic QTL again at top of chromosome 4, including cellobiose and propanoic acid [22].

**Figure 4:**
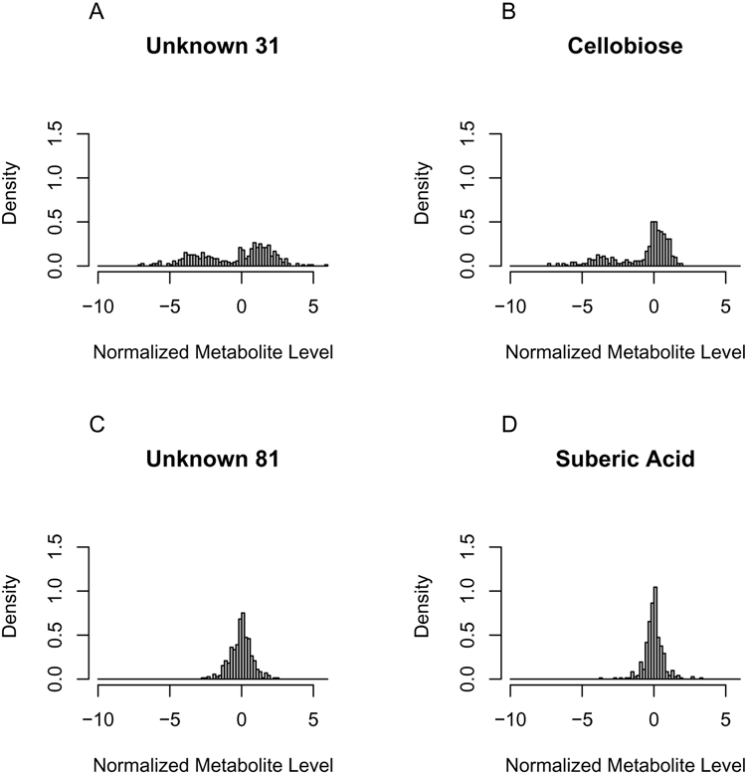
Histograms of particular metabolic markers over all 359 investigated RILs. Here, unit area histograms are presented, i.e. the particular curve shows proportions rather than absolute numbers. Thus it constitutes a simple density estimate. The x-axis demonstrates normalized metabolite levels and is divided into equidistant intervals. The y-axis represents the relative frequency per interval. The panels A and B show the two metabolic markers with the highest VIP in each investigated model, i.e. Unknown 31 (using a functional group prediction service offered by the Golm Metabolome Database [41] at least one hydroxyl group was predicted to be present in Unknown 31) and Cellobiose. The levels of these highly predictive metabolic markers deviate obviously from normal distributions, namely they display bimodal distributions. The deviation from a normal distribution seems to abate with decreasing importance of the particular metabolic marker in the models. This is demonstrated by the two examples C and D of metabolic markers, which have in average the lowest VIP in our models.

When comparing different metabolic markers concerning the distribution of their levels among the 359 RILs, it becomes obvious that the highly predictive markers tend to deviate from normal distributions (cf. Figure 4). The distribution is bimodal in the case of the most important metabolic markers, in other cases it is just too broad at the basis to pass for a single normal distribution. The deviation from a normal distribution seems to abate with decreasing importance of the metabolite in the prediction models (cf. Figure S1).

## Discussion

### Combination of genetic markers with metabolite markers leads to a significant improvement of heterosis prediction, which implies the existence of epistatic gene effects

As shown in the Results section, the combined use of selected genetic and metabolic variables of the RIL population leads to a significant improvement of the predictive power for the heterosis effect observed in the progeny of both test parents, i.e. the C24 and the Col-0 testcrosses.

The cause of this can at least partially be rationalised. It can be assumed that heterosis is primarily, if not exclusively, dependent on the match of the two alleles (or sets of alleles) derived from both parents, suggesting that complete genetic information should be sufficient for predicting heterosis. The genetic markers used here and in most other studies however are far from representing a complete coverage of the entire genome. On the other hand, in our study, individual genetic markers contain much information on the neighbouring regions, since we investigated a RIL population. Each line carries on average 1.86 recombinations per chromosome, and the average length of segments of a certain parental origin is 32.06 cM [23]. As illustrated by the slow decay of linkage disequilibrium among markers, the information carried by individual markers decreases only slowly with increasing distance. Consequently, strong individual gene effects, which are independent of other genes, should be reflected by our genetic markers, i.e. their effects should be recognised as effects of the marker alleles closely linked in coupling. However, in those cases where many genes are involved, genetic markers will not provide much information, especially if each gene contributes only a minor effect, which, in addition is depending on other genes (i.e. when epistasis has a high influence). In this situation, more integrated markers, such as metabolite levels, will contribute a decisive part of information, since they reflect the combined effect of many genes.

In our analysis, metabolic markers turned out to carry information on heterosis that is complementary to the genetic markers' information. Therefore, one should assume that epistatic gene effects are actually involved in heterosis. This is consistent with recent analyses which indicate a strong role for epistasis in the manifestation of heterosis in *Arabidopsis* [24], [25].

**Figure 5:**
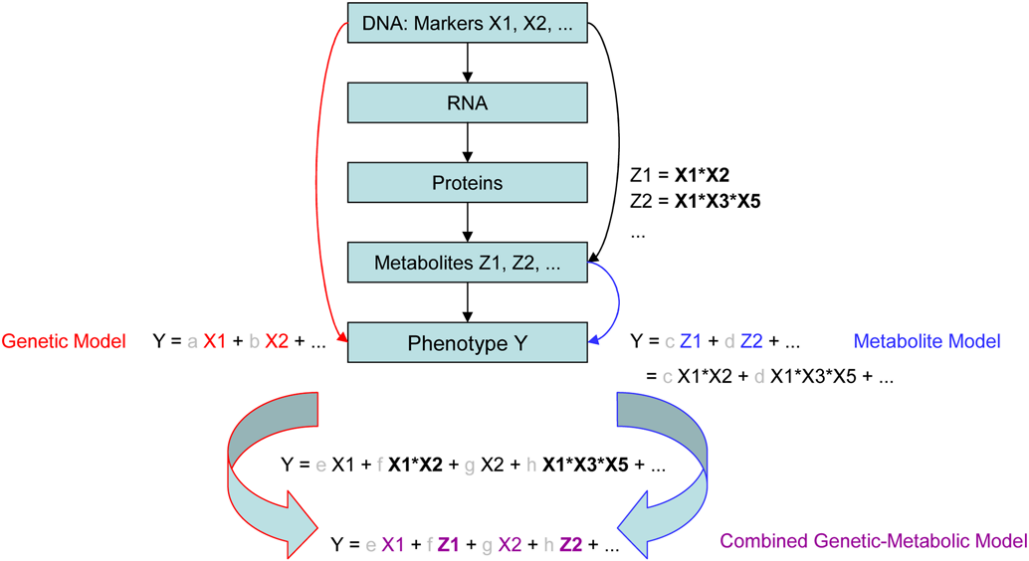
Simplified display of the idea behind the modelling for the different predictor sets. The chain of causality from genes to phenotype is displayed here. Since genes are at the starting point of the causal chain, one established way to model a phenotype Y is to use genetic markers X and combine them linearely (genetic model in red). Using instead predictor variables Z, which are close to the phenotype, such as metabolites, presents another promising way to predict a complex phenotype, since those variables integrate already parts of the complex gene interactions (represented by products Xi*Xj). The advantage is that we do not need to know, which genes actually interact in which way, and that the model can stay simple. It just linearely combines metabolite variables, thus integrating non-linear interactions indirectly (metabolite model in blue). In the end, one might use a combination of both approaches, i.e. combining different levels of the causal chain, to explain as much as possible of the complex phenotype. Hence one integrates linear relationships concerning the response as well as non-linear gene interactions, while sticking to the simple model ansatz of a linear combination of genetic predictors X and metabolite variables Z (combined model in violet).

Concerning an effective genetic heterosis prediction model, the complex trait thus would require to be modelled via complex nonlinear interactions of the different genetic markers. Since we know little concerning gene-gene interactions in relation to their influence on the trait of our interest, too many possibilities exist for modelling such a nonlinear phenomenon. Thus, nonlinear gene interactions should be integrated indirectly by using metabolic markers. This is very simple to model, since this approach essentially follows a linear combination of genetic and metabolic markers (cf. Figure 5). Nevertheless, this simple combined model appears to cover at least some parts of the complex gene-gene interactions, which are not yet captured by linear combinations of the available genetic markers, indicated by its significantly increased predictive power.

### Parental biomass, as an even more integrated variable than metabolite levels, is a strong but not a steady predictor for heterosis

Biomass is an integrated variable reflecting the action (and thus presence) of many, if not all, genes of the respective genotype. We therefore expected, following the argument used for metabolic markers, that the RILs' biomass as additional predictor is even more valuable.

Actually, just like metabolic markers (cf. Table 1), the RILs' biomass alone holds some considerable information on heterosis. Namely, RILs with a low biomass tend to have a higher potential for heterosis effects and vice versa, which is indicated by a negative coefficient of the biomass variable in the corresponding prediction models. This tendency is weaker in the case of Col-heterosis than in the case of C24-heterosis, as the predictive power of the pure biomass model is 0.44 for C24-heterosis and 0.35 for Col-heterosis in LOOV.

The combination of the RILs' metabolic markers and the biomass variable as predictors yields a predictive power of 0.51 and 0.47 for C24- and for Col-heterosis respectively. These numbers correspond approximately to the predictive power of the combined metabolite-genetic models (cf. Table S1). Furthermore the number of essential variables is massively reduced in the metabolite-biomass models as compared to the metabolite-genetic models (cf. Table S2). Hence, in combination with metabolite measurements, the biomass variable is more efficient than the genetic markers.

On the other hand, we added the genetic markers as predictors to the pure biomass model. In that way, the predictive power has been increased in both testcross populations to an almost equal amount, from 0.44 and 0.35 up to 0.60 and 0.50 respectively, for the C24 and the Col-0 testcrosses, independently of the presence or absence of metabolic markers as predictors. In both testcross populations, these numbers represent a significant improvement, as compared to all considered models (cf. Table S1). But because of the difference of predictive power in the two testcross populations, which depends clearly on the different predictive power of the biomass variable itself, the latter is - though a strong - but not a steady predictor for heterosis according to different accessions.

### Dominance effects as an explanation for further differences in C24 and Col-0 models

Some of the predictors, such as parental biomass, are significantly correlated to the heterosis effect in at least one of the testcross populations. Accordingly, their contribution in the respective multivariate models is high. Such variables show an interesting difference in their influence on heterosis prediction in the two testcross populations.

In contrast to the biomass variable, which correlates stronger with C24-heterosis (Pearson correlation: −0.45) than with Col-heterosis (Pearson correlation: −0.36), the remaining highly predictive markers are more important for Col- than for C24-heterosis. The most important single effect is that of the first genetic marker at top of chromosome 4. Its correlation with Col-heterosis is 0.40, as compared to 0.17 with C24-heterosis. The four subsequent markers on top of chromosome 4 also have a higher single effect on Col-heterosis (correlations between 0.31 and 0.35) than any marker has on C24-heterosis, where the strongest correlation (apart from the biomass variable) is −0.20 with a specific marker in the middle of chromosome 3.

Since the most important single effects are stronger on Col-heterosis than on C24-heterosis, the contribution of the corresponding markers in the multivariate genetic model is also higher in the case of Col-heterosis (cf. Figure S2). Here a small number of highly predictive markers is already sufficient to describe the response. In consequence, the minimal genetic prediction model for C24-heterosis contains much more variables than the corresponding Col-heterosis model (cf. Table S2).

Naturally, this affects also the combined genetic-metabolic models (cf. Table S2), not only because the most important metabolic markers also have a slightly stronger effect on Col-heterosis, but above all because in the Col-0 model the incorporation of the marker at top of chromosome 4 boosts the predictive power in one step, whereas in the C24 model many less important markers are necessary for the same increase (cf. Figure 2).

These observations about the substantial influence of top of chromosome 4 for Col-heterosis (VIP-ranking 1 in the minimal genetic model) in connection with its minor influence on C24-heterosis (VIP-ranking 22 in the minimal genetic model) can be explained in the following way. It is crucial for strong Col-heterosis that the respective testcross is heterozygous at top of chromosome 4, i.e. that the corresponding parental RIL carries C24 alleles at this locus, indicated by a positive coefficient of the marker in the model (cf. Methods section for the coding of the genetic data). For C24 testcrosses it does not matter (as much) which allele is present at this special genetic region. This fact suggests that C24 has a (partial) dominance effect at top of chromosome 4. We assume the dominance effect to be a partial one [26], [27], since in the C24 model the presence of C24 alleles of the corresponding RIL parent has still a slightly significant positive effect on the degree of heterosis. This is confirmed by the detection of a biomass QTL with a partially dominant effect in this region (cf. Meyer *et al.* 2008 submitted).

We found an opposite trend for the genetic marker in the middle of chromosome 3, which is only important for C24-heterosis (VIP-ranking 5 in the minimal genetic model). Indicated by the negative sign of the coefficient, the respective RIL parent is required to carry Col-0 alleles at this locus to yield high heterosis in C24 testcrosses, whereas it does not matter for Col-0 testcrosses whether they are homozygous or heterozygous at this locus. Consequently Col-0 is assumed to be dominant at the middle of chromosome 3. The same rational is applicable for some other accession-specific markers at other loci. Nevertheless, as mentioned above, we have to take into account also epistatic effects, in consequence of which the single dominance effects are often not as clearly revealed.

### The metabolic balance concept indicated by metabolic markers with inverse contribution to C24- and Col-heterosis prediction

Concerning the metabolic markers important for C24- and Col-heterosis prediction, there are some of them, whose model coefficients are positive in the Col-0 model while negative in the corresponding C24-model (e.g. glycine) and vice versa. This means, for instance in the case of glycine, that high levels in the parental RIL are associated with high Col-heterosis but with low heterosis in the C24 testcross. On the other hand, we find in the Col-0 parent a low level of glycine, whereas it is high in the C24 parent (and moderate in the F1 progeny). Consequently, the fact that a Col-0 testcross shows stronger heterosis when the corresponding RIL parent has a high glycine level, and a C24 testcross performs better when the corresponding RIL parent has a low glycine level, suggests that heterosis is dependent on an optimally balanced level of certain metabolites.

That is in agreement with the established metabolic balance concept of heterosis, which requires the coordination of all reactions and systems for efficient growth under a given environment [28], [29]. Even earlier a better metabolic balance in the hybrids [30] or metabolic control of fluxes [31] has been proposed as possible mechanisms for heterosis.

This idea is also supported by some of our specific metabolic markers (cf. Table 2), whose VIP-ranking is high in one testcross setting, while low in the other one. For instance the salicylic acid marker is strongly contributing to C24-heterosis prediction (VIP-ranking on position 7), but of minor importance in our models for predicting Col-heterosis (cf. Tables S4 and S5). Interestingly, the level of salicylic acid again differs in the parental accessions C24 (high level) and Col-0 (low level). The coefficient for this metabolic marker in the C24-heterosis models is negative, which indicates higher heterosis potential of RILs with lower salicylic acid levels. This suggests that the salicylic acid level must not be too high in at least one parent of a well performing testcross. It could be interpreted in such a way that too high alertness of the plant with respect to pathogens (as indicated by too high salicylic acid levels) draws too much of the metabolism into defense associated pathways, thus leading to suboptimal growth.

### Qualitative prediction of heterosis is possible using the combined genetic-metabolic marker set detected by our quantitative approach

As described in the Results section, combined genetic-metabolic models yield a correlation of approximately 0.5 between observed and in LOOV predicted response (cf. Table 1). Although this is a highly significant correlation, as proven by the fact that none of 1000 random permutations of the observed response showed such a high correlation to the predicted response, the respective prediction models cannot be used for reliable quantitative predictions.

However, an enrichment of crosses displaying a higher likelihood for strong heterosis would probably be helpful. To evaluate whether this approach might be feasible, we trained a linear discriminant model for C24-heterosis. This model discriminates the 359 RILs into lines with high and low heterotic potential using our minimal genetic-metabolic predictor set. In LOOV 233 RILs (i.e., 65%) were classified correctly by that model. Incorporating all 181 metabolic markers and all 110 genetic markers resulted in a discriminant model that classified only 49% of the RILs correctly, which corresponds to a random classifier. Thus the two-step approach described here, i.e. identifying in a quantitative model the most important set of genetic and metabolic markers and subsequently applying those in a qualitative model, might be an interesting path also with respect to practical applications [32].

In addition to the incorporation of gene interactions by metabolic markers, as shown in this study, we will also consider nonlinear relationships of the most important metabolic markers identified in this work. This seems to be a promising step, since on the one hand, the highly predictive metabolic markers show distributions deviating from normal ones. On the other hand, for linear models normal distributions of the predictors are required. Consequently, the consideration of some nonlinear terms in the discrimination model, which account for the special distributions of the highly predictive markers, will probably improve the classification success.

## Materials and Methods

### Data and software

Data was derived from a RIL population [23] within the framework of *Arabidopsis thaliana* heterosis experiments that included the accessions C24 and Col-0 [17]. We performed a comparative study on a set of 359 RILs and the two corresponding sets of testcrosses. All variables of interest were available for each of these 1077 lines, and they are described below. The mean frequency of C24 alleles and Col-0 alleles per RIL was 48% and 50%, respectively (cf. Figure S3). In average, 2% of the markers were heterozygous.

We investigated two sets of predictor variables, both, independently and in combination. The first set was the metabolic profile of the RILs consisting of 181 metabolic markers. Their levels were measured via gas-chromatography/mass-spectrometry at 15 days after sowing, and the data was normalized as described elsewhere [19]. Close to one half of the metabolic compounds were identified and 98 substances remained unknown but could at least partly be classified. The second predictor set was composed of 110 DNA-markers distributed along the whole RIL genome [18]. The available genetic marker information had been coded in the following way. Depending on whether the particular DNA-marker featured C24 or Col-0 it was quantified with 1 or 0 while markers with a heterozygous specificity were translated into 0.5. Single missing values have been replaced by the average of the direct neighboured markers' values in that RIL. If the missing value was at the margin, it just has been replaced by its single neighbour. Markers with more than ten per cent missing values had been deleted in advance.

Our response variables of interest were C24-heterosis and Col-heterosis. These were determined by the shoot biomass of the particular RIL, the shoot biomass of the corresponding testcross C24 x RIL (or Col-0 x RIL) and that of the background line C24 (or Col-0). Shoot biomass was measured as dry weight at 15 days after sowing. The determination of the shoot biomass values (cf. Table S6) via mixed model has been described elsewhere [17].

The absolute mid-parent heterosis is defined as the difference between the hybrid's shoot biomass and the mean shoot biomass of its parents. Dividing the absolute mid-parent heterosis by the mean shoot biomass of the parents, results in the relative mid-parent heterosis, which we focus on in this analysis (cf. Figure 1). In our case, the first parent is C24 (or Col-0) and the second parent is one of the 359 RILs.

Our computations were conducted on the free software environment *R* for statistical computing and graphics [33] in the version 2.5.1. Especially, we used the *R* package *pls* [34] in the version 2.0-1.

### Latent variables

For training the particular prediction models, we used the PLS approach [20], [35], which is a multiple linear regression method that works on latent variables. It projects the original predictor data into a latent lower dimensional space and thus maximizes the covariance with the response variable. The corresponding *R* function within the *pls* package is called *plsr* [34]. We realise the optimization according to the number of latent variables via LOOV (i.e. N-fold cross-validation with N = 359) of the Pearson correlation between the observed and predicted response. This is achieved in *R* by setting the parameter *validation* of the function *plsr* to “LOO”. The correlation, achieved with the optimal number of latent variables, is referred to, in this work, as the predictive power of the considered set of predictors for the response of interest. The *R* function to accomplish these computations is called *plsr*.

The rational of using PLS instead of ordinary least squares regression is the potential existence of latent variables, on which the particular response depends, but which are not available, because they cannot be measured. Fortunately, one latent variable often regulates not only the response of interest but also several other variables, which are in fact measured in experiments. Since they are all influenced by the one latent variable, many of them correlate strongly. Therefore we can down-weight some of the measured variables. The remaining variables could be considered as biomarkers for the particular response. That means there is not necessarily a causal dependence, but the biomarkers act as an indicator for the response. Since we consider very complex response effects, such as heterosis, probably several latent variables are involved. Thus, it is possible that two different latent variables influence the same measurable variable. For example, one of them up- and the other one down-regulates the measurable variable. Then, knowing only the value of the measured variable, it is not possible to reconstruct both latent variables correctly. We therefore have to weight the measured variables and to select the optimal number of latent variables. We use the PLS-algorithm for this purpose. Low weights in important latent variables give a hint for measured variables that do not improve the prediction.

### Feature selection

The PLS-approach is able to handle a high number of measured predictor variables relative to the number of samples. But certain response effects are very sensitive with regard to overfitting. Down-weighting the measured variables with a low contribution to the response, is not enough in this case. We therefore go one step further and suppress variables that contribute the least to the response of interest in the PLS-model.

The contribution of a predictor variable to the response is measured by the VIP suggested previously [20], [21], i.e. the variable's importance in the projection of the data into such a space of latent variables, which corresponds best to the response. We rank the original predictor variables with respect to their VIP. Afterwards, low ranked variables are deleted from the model. In contrast to other methods [32], we do not use a fixed cut-off criterion. Instead, we select such a number of original variables, which yields the highest predictive power in a new refitted PLS-model. Thus, we run a nested optimization loop. The optimization on the higher nested scope is according to the number of original variables, whereas the optimization on the lower nested scope is subject to the number of latent variables.

The computational costs of optimizing the number of latent variables increase with the number of original variables in the model. We therefore do not undertake those efforts for every possible number of original variables. The increment of our outer loop is ten, as a start, to get a general idea (cf. Figure S4 A). Afterwards, we reboot the outer loop with a refined increment of one, but break it after surpassing the two best arguments of the first boot by twenty steps (cf. Figure S4 B). This secures the identification of the optimal number of original variables, but it spares the refined search in unpromising regions with unjustified computational costs.

### Significance test

To exclude the possibility that the predictive power is insignificant (which means that the in LOOV predicted response does not correlate significantly with the observed response), we perform a randomisation test. We build 1000 random permutations of the true response. Then the Pearson correlation between the in LOOV predicted response and each permutation is calculated. The 1000 random correlations are compared to the predictive power, i.e. the correlation between the predicted response and the real response.

### Confidence intervals

To judge the difference in predictive power between two distinct predictor sets, confidence intervals for the predictive power of optimized models are estimated using jackknife resampling. Given a predictor set and a response variable, we randomly select 358 out of our 359 samples and recompute the predictive power of the optimal variable selection on the resampled jackknife data. The resampling and the recomputation of predictive power are repeated 200 times. This results in a specific range of values for the predictive power of the regarded optimal variable selection for the particular response. We consider the central 99% of those 200 values as an estimation for the confidence interval. It stretches from the mean of the two smallest values to the mean of the two largest ones. If the estimated confidence intervals for different predictor sets or for different response variables are disjoint, we consider the discrepancy of the corresponding predictive powers significant.

### Occam's razor

In addition, the estimation of a confidence interval for the optimal predictive power enables us to follow Occam's razor [36]. This widely approved principle denotes the simplest model, which contains the least variables, as the best among similarly descriptive models. The MDL [37] and the Bayesian Information Criterion [38] are just two examples that implement this principle. Since modern technologies, such as gene expression chips, confront us with a vast number of measured variables rising above the number of samples, it has become crucial, in recent years, to find a compromise between the acceptable loss of information and the desired number of variables [39]. In our approach, we use the VIP-ranking and the confidence interval to implement the principle. We look for the minimal number of highly ranked variables, whose predictive power is still located within the confidence interval estimated for the predictive power of the optimal variable selection (cf. Figure S4 C). We refer to the corresponding set of variables as the minimal variable selection.

### Response-specific predictors

The predictor variables, which are always contained in the minimal variable selection, can be considered as biomarkers. Comparing the biomarkers for the two different testcross settings, we learn about common and specific effects, We refer to a certain metabolic marker as specific for heterosis prediction in one testcross population, if and only if it is contained in both, the minimal metabolic variable selection and the minimal variable selection of the combined genetic-metabolic approach for one testcross setting, but in none of the corresponding minimal variable selections for the other testcross setting. On the other hand, we consider a certain metabolic marker as highly predictive for heterosis in both testcross populations, if it is contained in the minimal metabolic and also in the minimal metabolic-genetic variable selection for both testcross settings (cf. Table 2). Analogous definitions can be applied to genetic markers.

If we want to consider the order of importance of the biomarkers (cf. Tables S4 and S5), we consult their VIP in the corresponding minimal variable selections, which is in general slightly different from the VIP in the original PLS-model that included all measured predictor variables.

## Supporting Information

Figure S1 Additional histograms of metabolic markers over the 359 RILs, in comparison to a random generation. The panels A-F show the six metabolic markers most important according to their VIP in the minimal metabolic and in the minimal combined genetic-metabolic models. Their deviation from a single normal distribution seems to abate with decreasing importance. Panels G-K show the 5 metabolic markers with the lowest mean VIP in the complete metabolic and the complete combined genetic-metabolic model for C24- and Col-heterosis, in comparison to a random generation of 359 numbers following a normal distribution with expectation zero and a standard deviation of one third (L).
(0.05 MB PDF)

Figure S2 VIP of genetic markers in the complete (A) and in the minimal (B) genetic models. The figure shows the contribution of the 110 genetic markers, which have been arranged horizontally, to the respective response in the different models. The darker the bar, the higher the VIP of the corresponding marker in the particular model. Panel A shows that, when training on the whole set of markers, the contribution to C24-heterosis prediction is more diffused on many different markers, whereas it is strongly concentrated on top of chromosome 4 in the case of Col-heterosis. Panel B shows the VIP when trained on those selected genetic markers, which turned out to be essential predictors in the respective models. Each of the five chromosomes is represented by at least one marker in both minimal models, which overlap on the chromosome 1, 3 and 4. The two markers at the bottom of chromosome 3 only selected as predictors in the minimal Col-heterosis model were confirmed to be Col-heterosis specific markers by the combined genetic-metabolic model (cf. Methods section for details and Figure 3). This holds also true for at least one marker on each chromosome in the case of C24-heterosis.
(0.11 MB TIF)

Figure S3 Histograms of allele frequencies. The figure shows how the allele frequencies are distributed in the RIL population. The panels A and B deal with the frequency of C24 alleles and Col-0 alleles, respectively. For each RIL there are 110 genetic markers featuring C24, Col-0 or heterozygosity. The x-axis represents the number of the corresponding alleles per RIL. The y-axis presents how many of the 359 RILs show the corresponding allele frequency. The mean frequency of C24 alleles and Col-0 alleles per RIL is 48% and 50%, respectively. In average, 2% of the markers are heterozygous. The minimal frequencies for C24 and Col-0 alleles are 11% and 10%, respectively, while the maximal frequencies are 89% and 85%. The standard deviation was about 15.4 and 15.1, respectively. Using a Mantel test (P<0.001) to estimate association between marker matrices, we did not find significant differences in marker distribution between the two sub-populations [22]. Distorted segregation ratios were detected at the bottom of chromosomes I and V, at the top of chromosomes III and IV and in the lower region of chromosome III [23].
(0.00 MB PDF)

Figure S4 Feature selection process in metabolite model for C24-heterosis. Panel A shows the crude optimization of predictive power according to the number of predictors in the model with increment 10. It determines the breakpoint for the refined version (panel B) to reduce the computational costs (cf. Methods section for details). In the end, the in panel B determined optimal number Opt of variables is used to estimate a confidence interval for the corresponding maximal predictive power. This can be seen in panel C, where black dots represent the predictive power of the optimal variable selection when models were trained on jackknife resamplings of the data. Their range determines the estimate of the confidence interval, which is represented by gray lines. Min is the smallest number of variables, whose predictive power is still within the estimated confidence interval of the maximal predictive power.
(0.03 MB PDF)

Table S1 Estimated confidence intervals of the optimal predictive power in leave-one-out validation.
(0.03 MB DOC)

Table S2 Number of variables in the optimal and minimal predictor sets, in comparison to the number of all available predictors in the corresponding set. Abbreviations: Het, heterosis; Gen, genetic marker set; Met, metabolic marker set; Bio, biomass marker; Gen-Met, combined genetic-metabolic marker set etc.
(0.04 MB DOC)

Table S3 List of metabolic markers that turned out to be relevant neither for C24- nor for Col-heterosis prediction in our analysis, i.e. omission of those markers did not significantly deplete the predictive power in the metabolic models or in the combined genetic-metabolic models.
(0.03 MB DOC)

Table S4 List of the 40 metabolic markers highly ranked in C24-heterosis prediction, sorted according to the sum of their VIP in the minimal metabolic (met) and in the minimal combined genetic-metabolic (gen-met) model. Interestingly, the three highest ranked metabolic markers are distinct from those most important for biomass per se prediction [17].
(0.02 MB XLS)

Table S5 List of the 40 metabolic markers highly ranked in Col-heterosis prediction, sorted according to the sum of their VIP in the minimal metabolic (met) and in the minimal combined genetic-metabolic (gen-met) model. Interestingly, again the three highest ranked metabolic markers are distinct from those most important for biomass per se prediction [17].
(0.03 MB XLS)

Table S6 Dry weight (DW) and rMPH values for the 359 investigated RILs and the corresponding testcrosses (TC-C24 and TC-Col), including the standard deviation (sd) and the arithmetic mean (mean) for each column.
(0.10 MB XLS)
