# Supplementary figures and images for: Improved Heterosis Prediction by Combining Information on DNA- and Metabolic Markers
Source: PLoS One. 2009 Apr 16;4(4):e5220. doi: 10.1371/journal.pone.0005220 (PMC2666157; doi:10.1371/journal.pone.0005220)

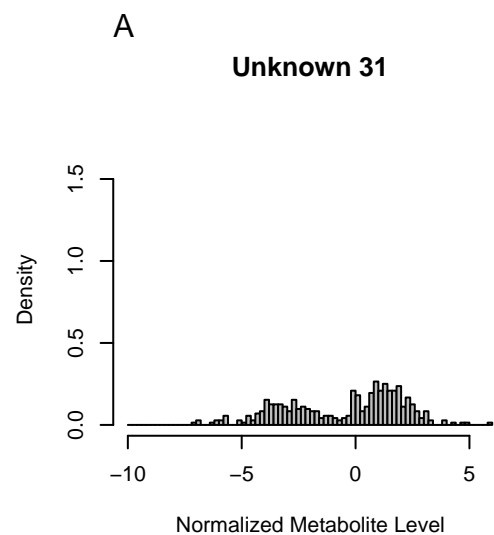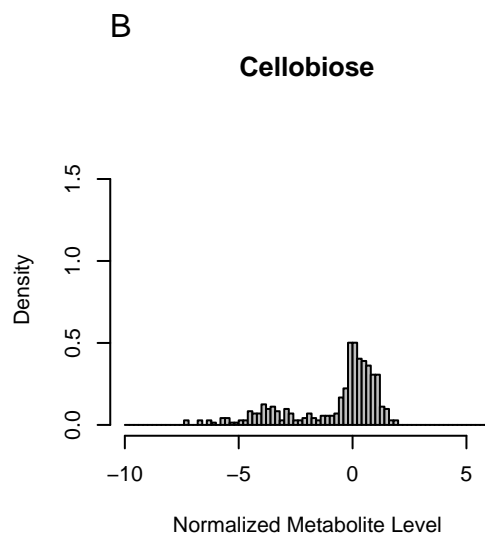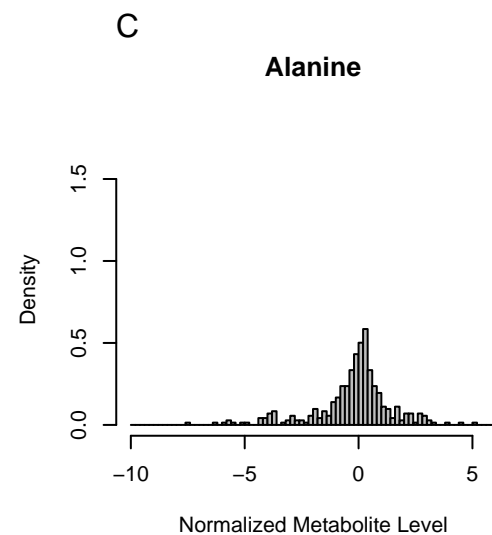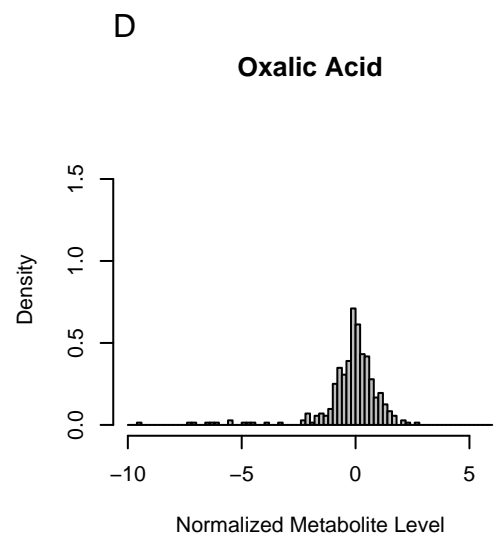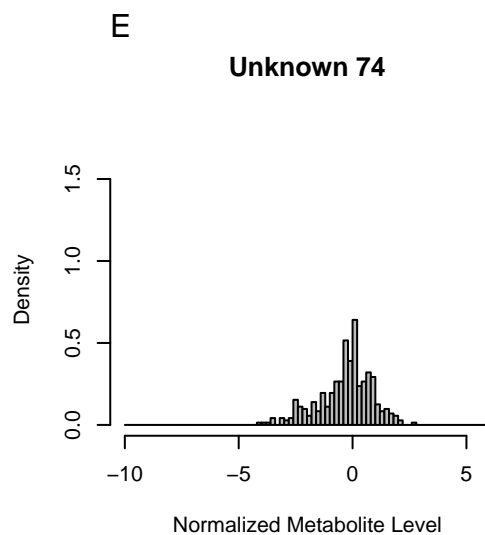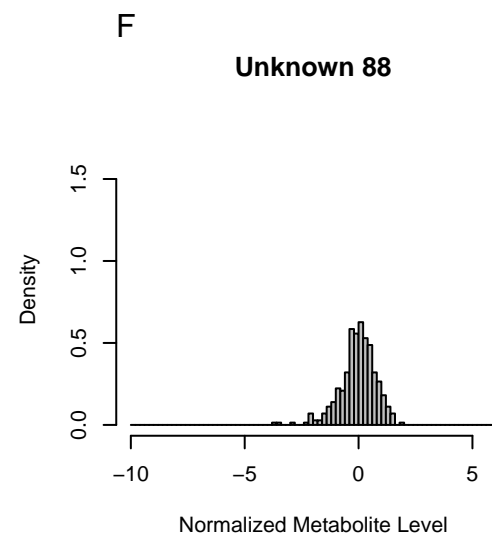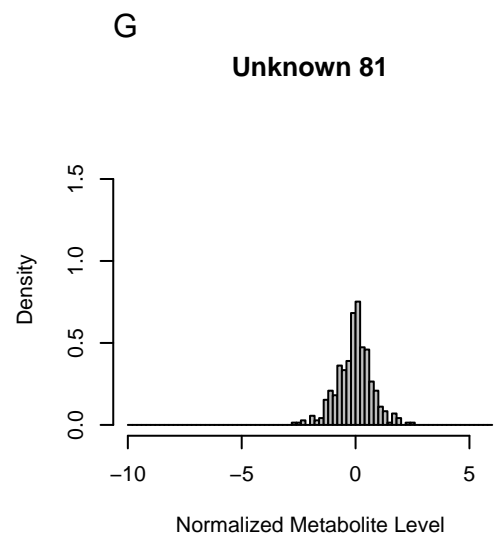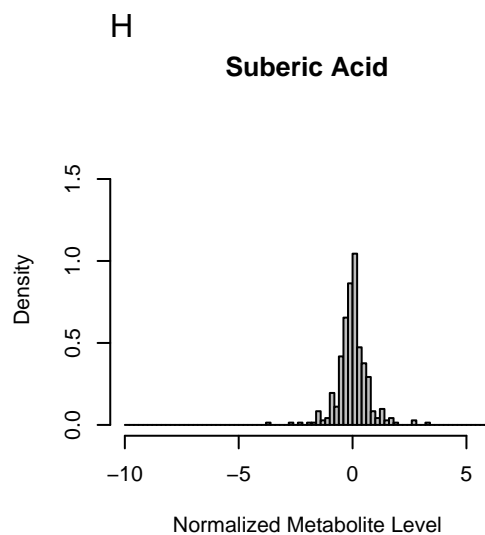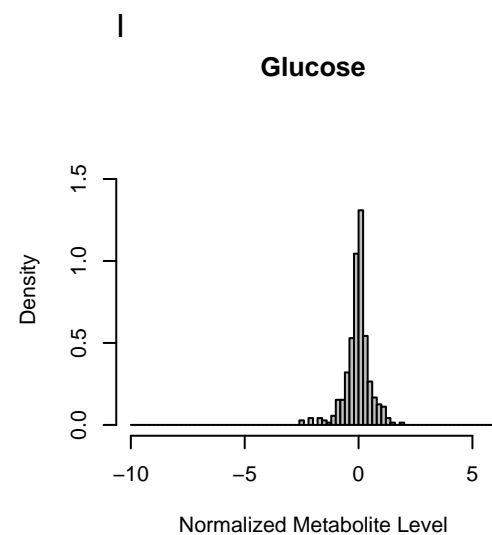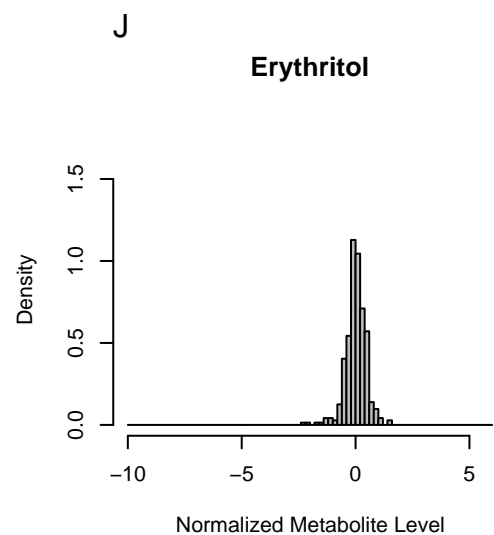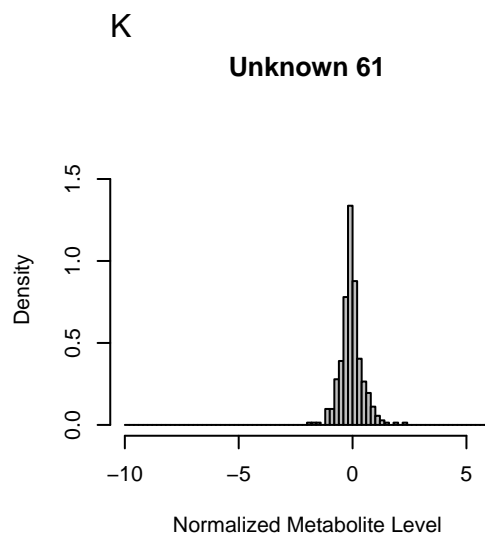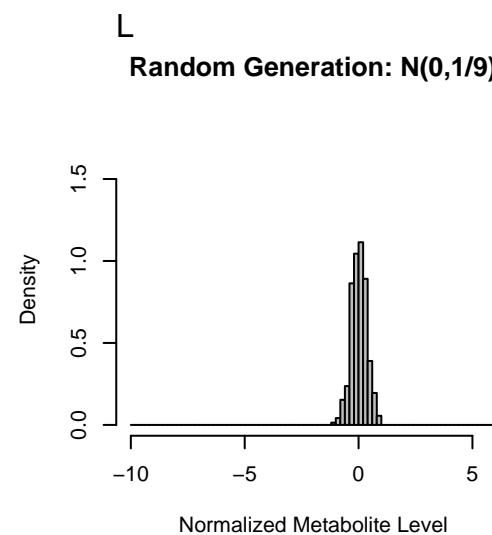

Supplement: Figure S1 — Additional histograms of metabolic markers over the 359 RILs, in comparison to a random generation. The panels A-F show the six metabolic markers most important according to their VIP in the minimal metabolic and in the minimal combined genetic-metabolic models. Their deviation from a single normal distribution seems to abate with decreasing importance. Panels G-K show the 5 metabolic markers with the lowest mean VIP in the complete metabolic and the complete combined genetic-metabolic model for C24- and Col-heterosis, in comparison to a random generation of 359 numbers following a normal distribution with expectation zero and a standard deviation of one third (L). (0.05 MB PDF) [file pone.0005220.s001.pdf]

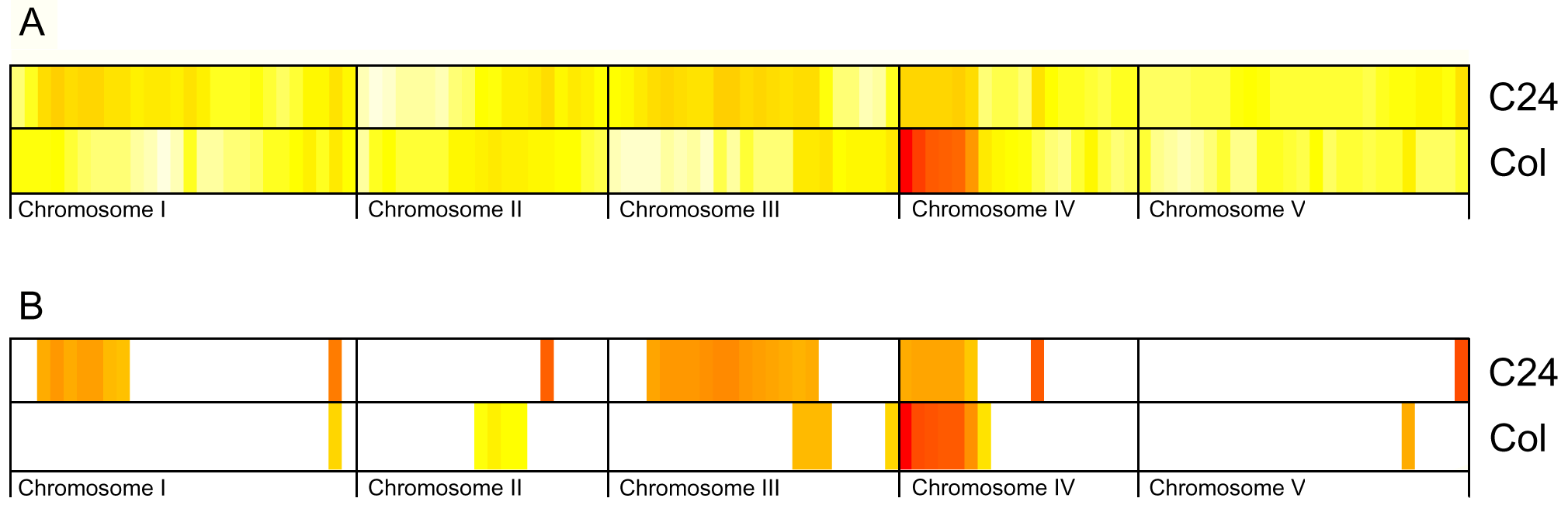

Supplement: Figure S2 — VIP of genetic markers in the complete (A) and in the minimal (B) genetic models. The figure shows the contribution of the 110 genetic markers, which have been arranged horizontally, to the respective response in the different models. The darker the bar, the higher the VIP of the corresponding marker in the particular model. Panel A shows that, when training on the whole set of markers, the contribution to C24-heterosis prediction is more diffused on many different markers, whereas it is strongly concentrated on top of chromosome 4 in the case of Col-heterosis. Panel B shows the VIP when trained on those selected genetic markers, which turned out to be essential predictors in the respective models. Each of the five chromosomes is represented by at least one marker in both minimal models, which overlap on the chromosome 1, 3 and 4. The two markers at the bottom of chromosome 3 only selected as predictors in the minimal Col-heterosis model were confirmed to be Col-heterosis specific markers by the combined genetic-metabolic model (cf. Methods section for details and Figure 3). This holds also true for at least one marker on each chromosome in the case of C24-heterosis. (0.11 MB TIF) [file pone.0005220.s002.tif]

A

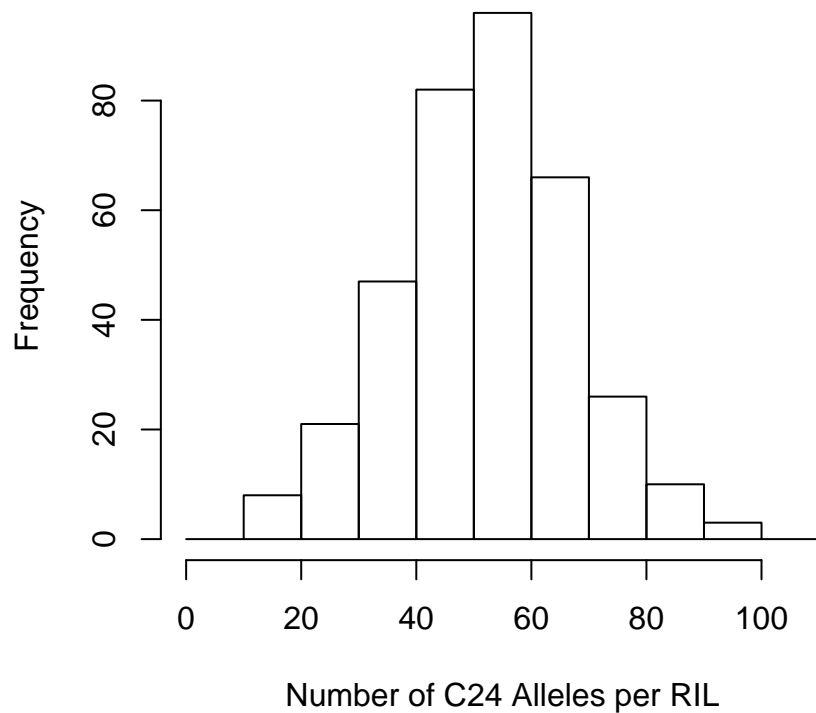

B

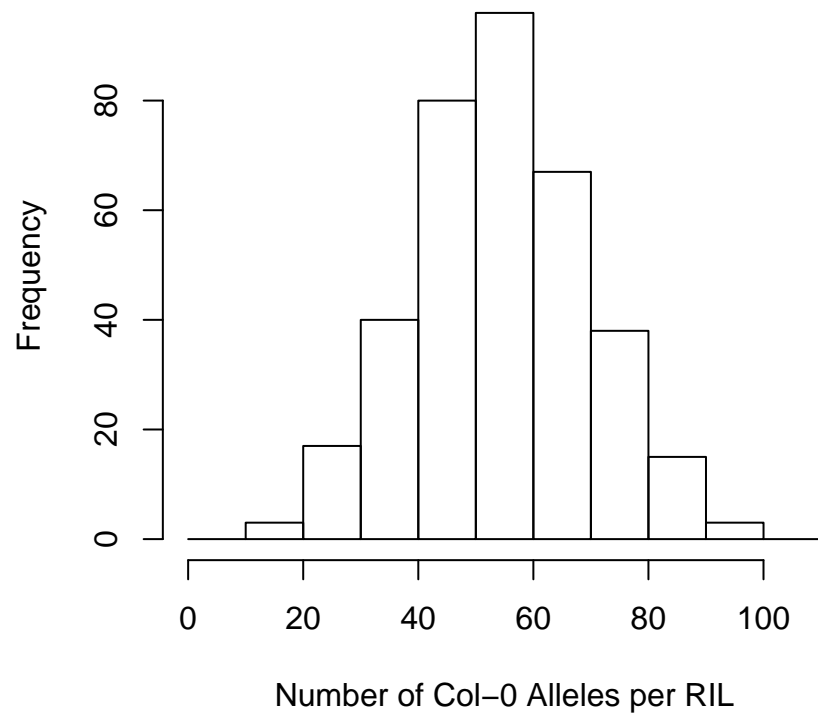

Supplement: Figure S3 — Histograms of allele frequencies. The figure shows how the allele frequencies are distributed in the RIL population. The panels A and B deal with the frequency of C24 alleles and Col-0 alleles, respectively. For each RIL there are 110 genetic markers featuring C24, Col-0 or heterozygosity. The x-axis represents the number of the corresponding alleles per RIL. The y-axis presents how many of the 359 RILs show the corresponding allele frequency. The mean frequency of C24 alleles and Col-0 alleles per RIL is 48% and 50%, respectively. In average, 2% of the markers are heterozygous. The minimal frequencies for C24 and Col-0 alleles are 11% and 10%, respectively, while the maximal frequencies are 89% and 85%. The standard deviation was about 15.4 and 15.1, respectively. Using a Mantel test (P<0.001) to estimate association between marker matrices, we did not find significant differences in marker distribution between the two sub-populations [22]. Distorted segregation ratios were detected at the bottom of chromosomes I and V, at the top of chromosomes III and IV and in the lower region of chromosome III [23]. (0.00 MB PDF) [file pone.0005220.s003.pdf]

A

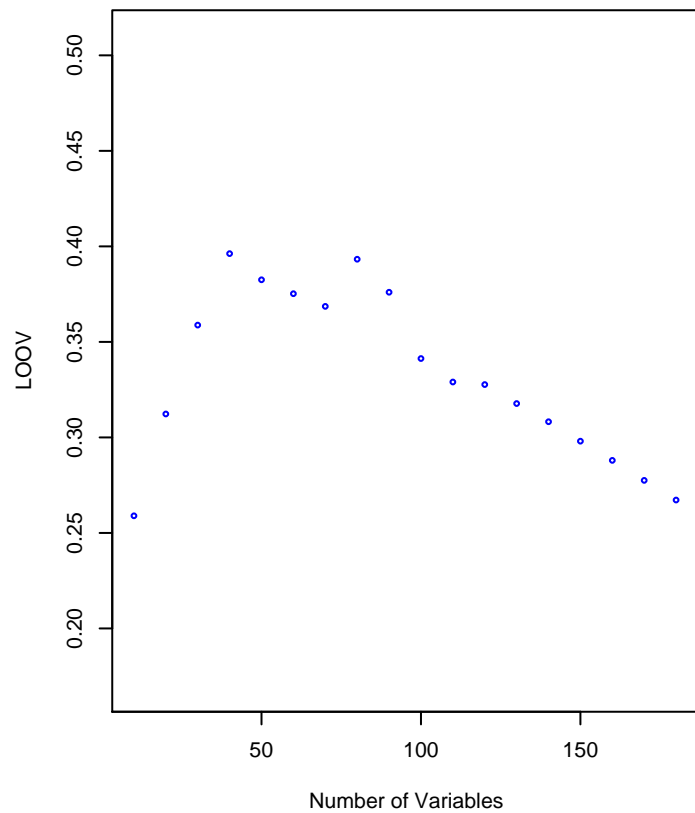

B

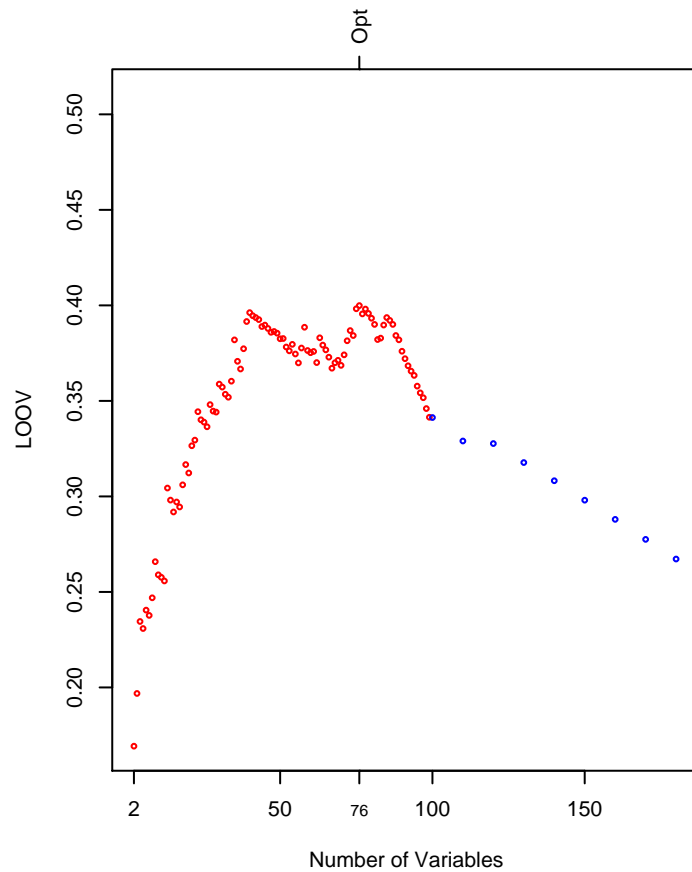

C

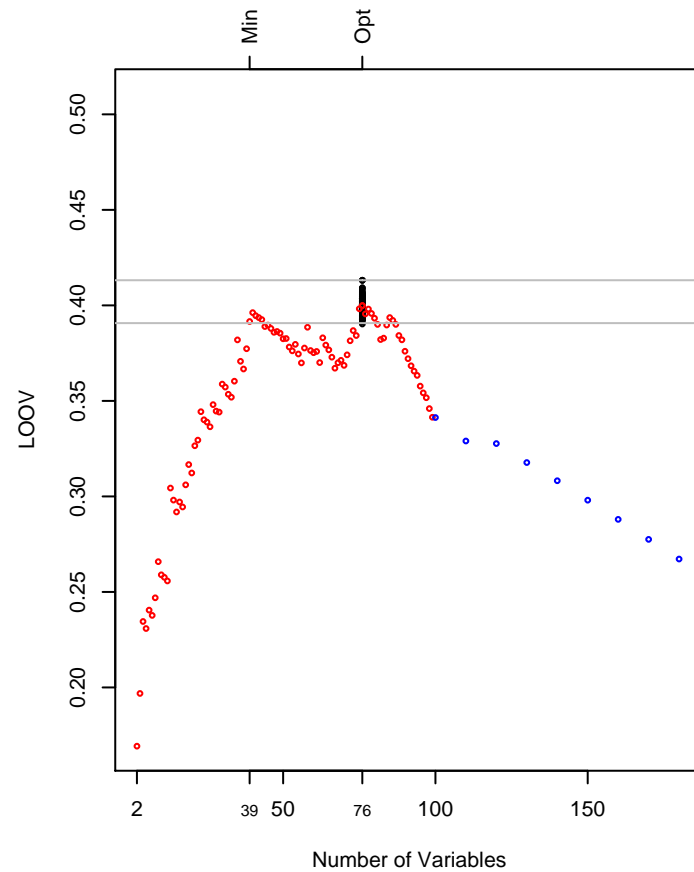

Supplement: Figure S4 — Feature selection process in metabolite model for C24-heterosis. Panel A shows the crude optimization of predictive power according to the number of predictors in the model with increment 10. It determines the breakpoint for the refined version (panel B) to reduce the computational costs (cf. Methods section for details). In the end, the in panel B determined optimal number Opt of variables is used to estimate a confidence interval for the corresponding maximal predictive power. This can be seen in panel C, where black dots represent the predictive power of the optimal variable selection when models were trained on jackknife resamplings of the data. Their range determines the estimate of the confidence interval, which is represented by gray lines. Min is the smallest number of variables, whose predictive power is still within the estimated confidence interval of the maximal predictive power. (0.03 MB PDF) [file pone.0005220.s004.pdf]
