# Supplementary material for: Improved Heterosis Prediction by Combining Information on DNA- and Metabolic Markers
Source: PLoS One. 2009 Apr 16;4(4):e5220. doi: 10.1371/journal.pone.0005220 (PMC2666157; doi:10.1371/journal.pone.0005220)
Supplement: Table S1 — Estimated confidence intervals of the optimal predictive power in leave-one-out validation. (0.03 MB DOC) [file pone.0005220.s005.doc]

| **Predictor set** | **C24-heterosis model** | **Col-heterosis model** |
| --- | --- | --- |
| Genetic markers | [0.4109, 0.4296] | [0.4086, 0.4240] |
| Metabolic markers | [0.3907, 0.4132] | [0.3649, 0.3902] |
| Combined genetic-metabolic markers | [0.4926, 0.5075] | [0.4760, 0.4949] |
| Combined biomass-genetic markers | [0.5962, 0.6147] | [0.4955, 0.5126] |
| Combined biomass-metabolic markers | [0.5079, 0.5217] | [0.4683, 0.4852] |
| Combined biomass-genetic-metabolic markers | [0.5889, 0.6051] | [0.4958, 0.5135] |
