# Supplementary material for: Improved Heterosis Prediction by Combining Information on DNA- and Metabolic Markers
Source: PLoS One. 2009 Apr 16;4(4):e5220. doi: 10.1371/journal.pone.0005220 (PMC2666157; doi:10.1371/journal.pone.0005220)
Supplement: Table S2 — Number of variables in the optimal and minimal predictor sets, in comparison to the number of all available predictors in the corresponding set. Abbreviations: Het, heterosis; Gen, genetic marker set; Met, metabolic marker set; Bio, biomass marker; Gen-Met, combined genetic-metabolic marker set etc. (0.04 MB DOC) [file pone.0005220.s006.doc]

|  |  | **C24-het model** | **Col-het model** | **C24-het model** | **Col-het model** |
| --- | --- | --- | --- | --- | --- |
| **Predictor set** | **complete** | **Optimal** | **optimal** | **Minimal** | **minimal** |
| Gen | 110 | 46 | 17 | 30 | 17 |
| Met | 181 | 76 | 33 | 39 | 30 |
| Gen-Met | 291 | 87 | 72 | 84 | 64 |
| Bio-Gen | 111 | 56 | 10 | 43 | 9 |
| Bio-Met | 182 | 42 | 31 | 42 | 31 |
| Bio-Gen-Met | 292 | 82 | 44 | 76 | 43 |
