# Supplementary material for: Improved Heterosis Prediction by Combining Information on DNA- and Metabolic Markers
Source: PLoS One. 2009 Apr 16;4(4):e5220. doi: 10.1371/journal.pone.0005220 (PMC2666157; doi:10.1371/journal.pone.0005220)
Supplement: Table S3 — List of metabolic markers that turned out to be relevant neither for C24- nor for Col-heterosis prediction in our analysis, i.e. omission of those markers did not significantly deplete the predictive power in the metabolic models or in the combined genetic-metabolic models. (0.03 MB DOC) [file pone.0005220.s007.doc]

| Adipic acid | Galactinol | Malic acid | Suberic acid |
| --- | --- | --- | --- |
| Azelaic acid | Glucose | Mannose | Threonic acid |
| Benzoic acid | Glutamic acid | Methanolphosphate | Threonine |
| Beta-Alanine | Glutamine | Methionine | Trehalose |
| Capric acid | Glutaric acid | Nicotinic acid | Valine |
| Citramalic acid | Glyceric acid | Ornithine | Xylitol |
| Citrulline | Glycerol | Phenylalanine | Xylose |
| Erythritol | Hydroxyproline | Phosphate | 55 Unknowns |
| Ethanolamine | Itaconic acid | Proline |  |
| Fructose | Leucine | Psicose |  |
| Fucose | Linolenic acid | Pyroglutamic acid |  |
| Fucosterol | Lysine | Sinapic acid (cis) |  |
